# Enhancing biodiversity conservation and monitoring in protected areas through efficient data management

**DOI:** 10.1007/s10661-023-11851-0

**Published:** 2023-12-05

**Authors:** Ferdinando Urbano, Ramona Viterbi, Luca Pedrotti, Enrico Vettorazzo, Cristina Movalli, Luca Corlatti

**Affiliations:** 1 European Commission, Joint Research Centre (JRC), Ispra, Italy; 2 Gran Paradiso National Park, Via Pio VII 9, 10135 Torino, Italy; 3 Stelvio National Park, Via De Simoni 42, 23032 Bormio, Italy; 4 Dolomiti Bellunesi National Park, Piazzale Zancanaro 1, 32032 Feltre, Italy; 5 Val Grande National Park, Piazza Pretorio 6, 28805 Vogogna, Italy; 6 Chair of Wildlife Ecology and Management, University of Freiburg, Tennenbacher Straße 4, 79106 Freiburg, Germany

**Keywords:** Applied ecology, Data curation, Ecoinformatics, National parks, Natura 2000, Natural reserves

## Abstract

A scientifically informed approach to decision-making is key to ensuring the sustainable management of ecosystems, especially in the light of increasing human pressure on habitats and species. Protected areas, with their long-term institutional mandate for biodiversity conservation, play an important role as data providers, for example, through the long-term monitoring of natural resources. However, poor data management often limits the use and reuse of this wealth of information. In this paper, we share lessons learned in managing long-term data from the Italian Alpine national parks. Our analysis and examples focus on specific issues faced by managers of protected areas, which partially differ from those faced by academic researchers, predominantly owing to different mission, governance, and temporal perspectives. Rigorous data quality control, the use of appropriate data management tools, and acquisition of the necessary skills remain the main obstacles. Common protocols for data collection offer great opportunities for the future, and complete recovery and documentation of time series is an urgent priority. Notably, before data can be shared, protected areas should improve their data management systems, a task that can be achieved only with adequate resources and a long-term vision. We suggest strategies that protected areas, funding agencies, and the scientific community can embrace to address these problems. The added value of our work lies in promoting engagement with managers of protected areas and in reporting and analysing their concrete requirements and problems, thereby contributing to the ongoing discussion on data management and sharing through a bottom-up approach.

**Supplementary Information:**

The online version contains supplementary material available at 10.1007/s10661-023-11851-0.

## Introduction

Understanding ecosystem complexity, temporal dynamics, and response to human disturbance is the basis of good conservation practices (Meffe et al., 2002). At the same time, the sustainable management of natural resources should embrace long-term ecological, social, and economic goals (UN, 2015; Van Dyke, 2008). A crucial role in biodiversity conservation is played by protected areas (Naughton-Treves et al., 2005; Pino-Del-Carpio et al., 2014), geographical spaces dedicated and managed to achieve long-term conservation of nature (Dudley, 2008). Over time, efforts on protected areas have progressively shifted from the traditional safeguarding of extraordinary sites or emblematic species to the management of a wide and complex range of ecological and social issues (O’Reilly & Murphy, 2010; Watson et al., 2014), supporting decision-making in the context of a broad, long-term public mission. This is especially relevant where conflicts between human presence and the conservation of natural resources have significant political implications (Davoli et al., 2022).

The acquisition of appropriate data-driven knowledge (Conde et al., 2019) is crucially important in this scientifically informed approach to the decision-making process (Grumbine, 1994) and in monitoring the management and policy impact against environmental indicators (ECA, 2020). Strengthening of scientific research and monitoring capacities, as well as the best available data, information, and knowledge being accessible to decision makers, is also integral part of the Kunming-Montreal Global Biodiversity Framework, to which most countries have recently committed (https://www.cbd.int/gbf/). The goals of data collection and management in protected areas often differ from those of other research institutions. They are based on monitoring of natural resources, appraisal of ecosystem services, promotion of cultural values, and support for conservation policies in connection with socio-economic activities and interests in areas under growing pressure from recreational activities (e.g. Beissinger et al., 2017). Data collected in protected areas may come from various sources, such as field surveys, camera traps, weather stations, animal tracking devices (e.g. GPS collars), high spatial and temporal resolution satellite images, molecular data, citizen science, and social media. Unfortunately, due to suboptimal data management, the vast amount of current and historical information collected by many protected areas is often affected by errors and inconsistencies and archived in a way that limits its reuse and compromises its long-term preservation (Zuckerberg et al., 2011).

The importance of data management for ecological research and biological conservation in the new age of information technology has been increasingly recognised (Applegate, 2015; Costello et al., 2018; Michener, 2016). Inappropriate data reporting, for example, may be a major source of research waste in ecology (Purgar et al., 2022). Key elements in securing the long-term availability of reliable information are biodiversity data accuracy (quality), security (protection against loss), documentation (compilation of metadata that describe the data and the protocols used to collect and store them), and accessibility. These objectives can be achieved through the use of appropriate tools and expertise (Chamanara et al., 2021; Hobern et al., 2014) and through implementing a data management cycle (Recknagel & Michener, 2018) that specifies how to handle data during collection, processing, documentation, and archiving, covering the whole data life cycle (Chamanara & König-Ries, 2014). This complex data landscape has given rise to a new discipline, ecoinformatics (Michener & Jones, 2012), within which concepts, practices, and challenges specific to biodiversity data have been developed (Gadelha et al., 2021). Moreover, to guide managers and researchers in the management and stewardship of scientific data and to support data sharing, clear principles such as findability, accessibility, interoperability, and reusability (FAIR) have been defined (Wilkinson et al., 2016). Nevertheless, and despite the consensus of the scientific community about the importance of reusing data (Reichman et al., 2011), it is estimated that only 10% of biodiversity data are available in digital form (Ball-Damerow et al., 2019). Those research groups and institutions, including most protected areas, that are known as the long tail of scientific research (Heidorn, 2008; Latif et al., 2019), still struggle to keep pace with the most advanced, best-funded, and skilled institutions (Hackett et al., 2019; Petters et al., 2019). Unfortunately, scientific literature provides few practical guidelines or protocols on data curation. This has resulted in these issues being given limited attention outside of the academic domain, representing an unfulfilled challenge for many conservation practitioners (Sarramia et al., 2022).

In this manuscript, we explore the main requirements of protected areas for data collection and management, based on findings from the coordinated activities carried out by the four Italian Alpine national parks (Gran Paradiso National Park, GPNP; Stelvio National Park, SNP; Val Grande National Park, VGNP; Dolomiti Bellunesi National Park, DBNP) since 2013. Protected areas may be considered an expression of human values, choices, and decisions (Rozzi et al., 2015), which makes any attempt to generalise difficult. Nevertheless most of the examples discussed here are commonly found in protected areas. Our aim is to highlight the critical points that, based on this experience, have been neglected in the mainstream discussion about data management and to suggest possible ways to address the specific requirements, opportunities, and challenges of protected areas.

## Materials and methods

### The collaboration among national parks in the Italian Alps

**Fig. 1:**
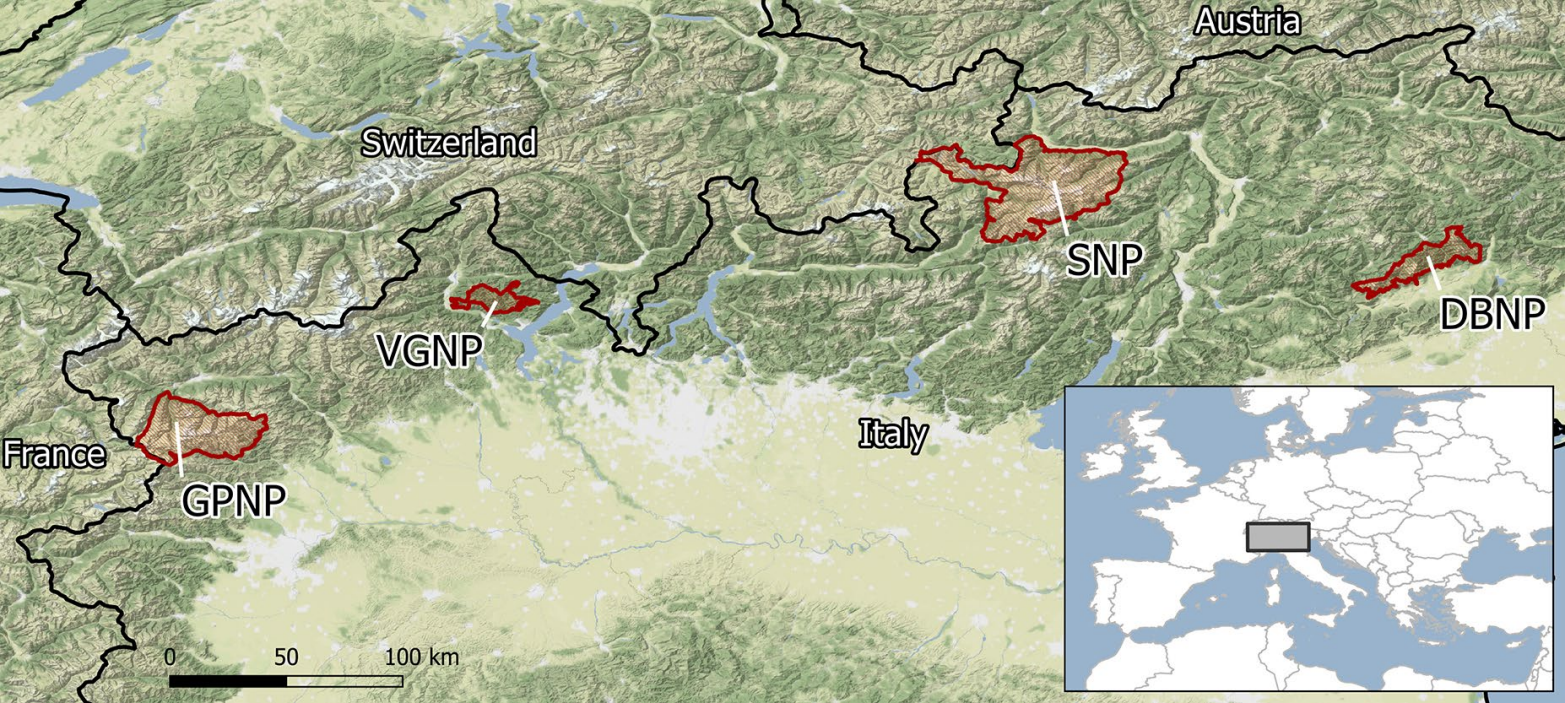
Location of the four national parks in the Italian Alps

The collaborative effort to improve data management in the four Italian national parks originated in 2013, when Gran Paradiso National Park realised that the collected data were not being fully harnessed for institutional reporting, research, or design of conservation plans, thereby limiting their potential impact. This was because procedures and tools for data management (e.g. comma-separated values files, spreadsheets, personal databases) did not offer appropriate solutions for effective and efficient data use. GPNP promoted a long-term initiative to improve its data management system, starting by creating a database for data collected under a long-term, shared monitoring activity, the Alpine Biodiversity Project (Box 1). The following year, Stelvio National Park (SNP) joined in with the same activity as a partner in the project. When structuring the data in a database, it became evident that it was not merely a problem of formatting and documentation. Despite common protocols, the complexity of data sets collected, transcribed, and processed by many people over many years introduced inconsistent, and at times erroneous, information that could only be partially detected by automated checks. Furthermore, much of the information was collected informally, as side notes, with limited analytical potential. Therefore, the two parks decided to scale up the biodiversity database to a more comprehensive information system to manage all the data they collected, some dating back to 1945. In 2018, the two other parks involved in the Alpine Biodiversity Project, Val Grande National Park (VGNP) and Dolomiti Bellunesi National Park (DBNP), joined the data management activity. The four parks started using the same quality check protocols and harmonised databases, thus including in this process all Italian Alpine national parks (see Additional Materials for a short environmental and administrative characterisation of the parks and Fig. 1 for a framing map). In turn, the project elicited stricter collaboration between the parks on defining a harmonised approach to the collection and management of current and historical data sets, extending the collaboration already established within the Alpine Biodiversity Project. This common effort resulted in four distinct repositories (one per park) where biodiversity data and other data sets are structured, documented, and quality checked, with protocols for their updates.

Box 1 The Alpine Biodiversity Project Mountain areas are sensitive biodiversity hotspots, being exposed to several threats, primarily related to human activities (such as livestock farming, agriculture, and tourism) as well as to global changes, including climate change and land cover changes (Terzi et al., 2019). Biodiversity monitoring is a priority to improve understanding of the evolutionary and mechanistic basis of ecological patterns in Alpine species distribution. The Alpine Biodiversity Project started in 2006 in Gran Paradiso National Park. From 2013, thanks to the Italian Ministry of the Environment, it was extended to a network of four national parks (Gran Paradiso, Stelvio, Val Grande, Dolomiti Bellunesi) and two regional parks (Alpi Cozie and Ossola) with the aim of creating a long-term shared monitoring programme. The Alpine Biodiversity Project was the first data set processed in the data management activity across the parks. Through surveys conducted over a variable number of circular plots located along several altitudinal transects, the main aim of the project is to investigate variations in spatio-temporal patterns of diversity in several core *taxa*, including *Lepidoptera Rhopalocera*, *birds*, and surface-active arthropods (*Coleoptera Carabidae*, *Coleoptera Staphylinidae*, *Orthoptera*, *Araneae, Formicidae*). Depending on the park it extends to other *taxa* such as amphibians and reptiles, *bats*, mesocarnivores, and rodents. Besides data on animal diversity, within each plot several environmental covariates are collected to characterise microclimatic conditions and micro-, meso-, and macro-habitat features.

### Methodological approach to data management

We started our work towards improving data management by compiling a catalogue of all the datasets collected by the four parks since their creation. We reviewed the tools in use, ranging from simple spreadsheets to personal databases. We assessed the quality of the data and identified the critical limitations of the data handling methods and protocols. This preliminary activity was the basis for the requirement analysis which, for each park, led to the design of an optimised data management approach built upon a spatial database with protocols for data acquisition, digitalisation, quality checking, and uploading. The information systems that implement this process were built using open-source software (PostgreSQL and PostGIS, an advanced spatially enabled database representing a state-of-the-art technical solution widely adopted by the scientific community). No specific interface was created, leaving database users with the choice to connect to the database to upload, visualise, and process data using their favourite tools, for example, spreadsheets, R (R Core Team, 2020), GIS tools, and Python.

The work on the Alpine Biodiversity Project data represented the starting point; it focused on defining a shared data structure for the database and on verifying and harmonising data reported by the parks. The data sets processed and integrated into the park databases were then extended to 25 other data sources, from GPS tracking devices on ungulates to field data on behavioural ecology and population dynamics of marmots, from camera traps to life-history traits and social interactions of individually marked ibex. They also included data sets on the abundance of ungulates (roe deer, red deer, chamois, ibex), wolves, mustelids, bats, and galliformes, as well as ranger observations.

When dealing with the historical data sets, additional processing steps were needed. Quality checks and documentation required a lot of operator-based assessment because the original records and the memory for the data collection stage were partially lost. Individual field paper forms had to be retrieved and analysed. Due to the very large data archives and the limitations to resources available, we processed only a subset (approximately 25%) of all surveys conducted in the parks, with a view to a complete archive recovery in the medium to long term.

The data management activities were carried out with the support of an external consultant to set up the database, implement a permission policy for the different users, and run automatic controls preliminary to the expert detailed screening. The consultant provided training to enable park staff to populate and use the database, and to familiarise them with the main concept and tools for efficient data curation. Special attention was given to the long-term sustainability of good data management practices beyond the scope of the project. All those responsible for data collection gained sufficient skills in data management to properly handle their data sets. Some of them also learned the database language, Structured Query Language (SQL), well enough to be able to update the data in the database and process and extract it for analysis. The purpose of having more skilled staff is also to provide technical support to less experienced colleagues in using the data stored in the database.

## Results

We illustrate the results in terms of data management challenges and solutions adopted while working on the recovery, control, harmonisation, documentation, and organisation of the data in structured repositories. There is only a partial overlap between critical issues in ongoing data collection and in historical data. For this reason, we discuss them in two separate sections. We start with describing the results related to processing of data collected in the past; then we report on the outcomes of the work for current data collection.

### Processing of historical data

Since their creation, the four parks have collected data on flora and fauna using different sampling designs and formats. Gran Paradiso National Park has collected data for 100 years, and Stelvio National Park for almost 90 years. Count series for ungulate species date back some 80 years. This has generated an enormous amount of valuable data for studying trends and responses to different pressures on the ecosystem (e.g. Parmesan, 2006; Rutz et al., 2020). This is particularly important because long-term biodiversity data are rare, and long-term series make it easier to disentangle the effects of ecological drivers. Such historical data, however, were often available only in their original format, as spreadsheets, comma-separated values files, or even paper sheets compiled in the field. In addition, the data collection protocols evolved over time, with older data often characterised by a lower level of formalisation (e.g. unstructured data, no use of controlled vocabulary, no extensive quality controls). The multiple operator-based steps performed in data transcription in the past make these data sets highly error-prone, ultimately resulting in low reliability of analysis results. We tried to recover as much information as possible, making it available in a format ready to be analysed and with an estimation of the uncertainty associated with it. This is also a preliminary step towards data sharing through presenting data in a standardised, and possibly machine-readable, format (outside the scope of the project).

#### Lack of documentation

One of the most challenging aspects of processing historical data was the poor documentation (metadata). A lack of data collectors’ historical memories and loss or damage to original field sheets necessitate a time-consuming, non-automatable, record-by-record check to resolve any erroneous, inconsistent, missing, or incomplete data.

#### Incorrect data

Incorrect data were primarily detected using semi-automated procedures by comparing data with acceptable ranges or sets of values. This identifies issues such as negative values of wind speed, age in years exceeding the biological limits of a given species, or body size reported in an incorrect unit of measurement (e.g. centimetres instead of metres). In some cases, potential outliers were identified using a statistical approach based on the distribution of values in the data sets. In other cases, valid ranges were based on expert knowledge of the species, its environment, and its behaviour. Date and time data types are poorly managed by flat file types such as spreadsheet or shapefiles, leading to frequent issues, for example, a mix of DD/MM/YYYY and MM/DD/YYYY formats caused by variation in local settings between computers. These problems were caught by observing the sequence of data recorded in the original format. Checking for duplicates of some sets of information, for example, the presence of a species in a plot on a specific date, helped to identify records with incorrect attributes. For some of the suspicious records detected, it was not possible to determine whether it was incorrect or due to exceptional conditions, such as the presence of an uncommon species for the site. In these cases if the data are true, they potentially contain highly significant information. For this reason, the original information has been kept and associated with quality codes (e.g. “reliable”, “low reliability”, “incomplete”) that can be used to include or exclude specific records during analyses. When data were modified and corrected, this was documented and the original value reported in the notes.

#### Inconsistent data

Another processing step was to detect inconsistencies, namely, the same piece of information being reported with different values in the data set. Frequent examples were the difference between the number of insects counted in a trap and the sum of individuals derived from species recognition, and the reference date of a transect survey compared with the date recorded in the individual observations. This type of control could be run only when data were collected with a certain level of redundancy.

#### Inhomogeneous use of classes

We also had to deal with an inhomogeneous use of classes, for example, to describe age, sex, weather conditions, or species name. The lack of controlled vocabulary and the evolution of class definitions over the years transformed these attributes into qualitative descriptions that could not be used consistently for analysis. This required a harmonisation process to remove errors such as inconsistent use of upper/lower case and spaces, typos, or equivalent expressions such as “very cloudy”, “many clouds”, and “cloud covered”. The recovery and management of species names were particularly challenging. This is due to different spellings, synonyms under different systems, evolution of recognised species, and integration of data collected by scientists with data collected from rangers (the latter often recorded using common names instead of scientific names). In this regard, an additional problem was the use of different taxonomic levels to identify individuals when the species was not always available because it was recognised at genus level (e.g. *Streptopelia*, *Chorthippus*), family (e.g. *Ixodidae*, *Hirundinidae*), or order (e.g. *Orthoptera* especially when found at larval state, *Chiroptera*). We used a flexible and consistent framework to track the taxonomic information to avoid forcing it into a single “species” attribute, mapping any species label (including common names) to its complete taxonomic description.

#### Multiple versions

Another problem that we faced for many data sets was the existence of multiple versions of the same file with different data cleaning performed on each of them, or information spread over different files with partial overlaps between them. This means that no version could be used as a reference, and specific and time-consuming work had to be done to derive a single, complete version.

#### Management of unknown data

The widespread ambiguity in unknown data being marked with 0 values instead of null required specific controls, especially in the census-related data sets where there is a significant difference between these two pieces of information. In some cases, it was not possible to determine whether the data were missing, or no individuals were observed. This was documented during screening by tagging the information with a specific reliability code. Another issue faced is the frequent use of a fictitious species (“no species”) to keep track of surveys with no species detected. This inefficient approach has been fixed using a proper data model where general information on the survey is separated from the observations.

#### Non-formalised information

We found another large class of problems when dealing with the notes, where many aspects of an observation were stored, limiting their potential use in the analysis. Challenging work was carried out to transform as much data as possible into distinct and searchable pieces of information. Another common issue was the use of personal comments such as generic question marks, coloured cells in a spreadsheet, or cryptic abbreviations that can be interpreted only by those who recorded them, leaving a high level of future uncertainty if not transformed into understandable information.

#### Spatial data

In the past, especially before GPS devices were used systematically, the location was recorded as points drawn on paper maps or with reference to local toponyms, often not reported on official cartography. In all these cases, it took a long time to digitalise the information. Another set of problems we encountered was the evolution over time of the spatial units used as reference (e.g. counting sectors, administrative boundaries). We kept all the original information and developed methods to aggregate/disaggregate according to specific criteria. The use of mixed coordinate reference systems also required additional work to harmonise and document them. Spatial data marked outside the areas of the parks needed additional screening to verify, where possible, whether the occurrence was actually observed there or they were just wrong coordinates.

### Processing of ongoing data collection

The initial focus of the project was to screen and integrate data collected through the joint Alpine Biodiversity Project (Viterbi et al., 2013) (see Box 1). However, many other data sets from current surveys were processed, providing a wide and diverse range of data management issues.

The work was carried out in strict collaboration with all the technicians responsible for data collection, using an interactive process. The data management consultant processed the data to identify the problems, then the park operators fixed all the issues, exploring the cause of errors and incompleteness. This led to substantial improvement in the quality of data reported from the field and to optimised data collection.

#### Complexity of data structure

The first element that emerged as potentially problematic was the complexity of the data collected. In ecology, the key biological standard is the species concept and traditionally the primary data are where and when species have occurred (Costello et al., 2018). Nevertheless, in recent years, monitoring projects have moved to more structured field campaigns where species occurrence is only part of the data collected. They also try to capture intra- and inter-species interactions and their relationships with the environment at multiple spatial and temporal levels. This includes information on population, communities or ecosystem dynamics, and adaptation of behaviour to changing conditions, along with data from sensors and genetic samples. This intricacy of data sets to be processed and stored clearly reveals the inadequacy of tools such as spreadsheets or personal databases commonly used by individual researchers. Effective tools to properly manage this complexity are spatial relational databases (Cushing et al., 2007). Other repository models exist, such as NoSQL databases, but these are less optimised for complex and structured ecological data (Sahatqija et al., 2018). Defining the data model needed for relational databases is an opportunity to formalise the key objects of a study and the relationship between them; this enables data consistency in the long run and improves identification of the information needed and how it must be collected (Urbano et al., 2010). The use of a relational database prevented incorrect data from being stored by forcing relational constraints and controls on data type and values. A robust database also allowed integration of sensor-based data (e.g. GPS tracking, animal-attached accelerometer, weather stations), and connection to remote-sensing products accessible in the cloud (e.g. Copernicus Sentinel-1 radar and Sentinel-2 optical satellite images).

#### Multi-user environment

Another major issue faced while handling data collected by ongoing surveys, typically involving many experts, is the need for a tool that can support a multi-user, remote environment providing a single version of the data. The original sets were replicated in several hard-to-synchronise versions which were passed from one expert to another, limiting the effectiveness of collaboration, slowing down the process, and jeopardising data quality. This issue was solved by adopting a centralised database, based on the open-source tools PostgreSQL and its spatial extension PostGIS.

#### Data quality

Unlike historical data sets, quality checks on data collected in recent or ongoing projects could rely on the controls by the technicians who went to the field, as the survey was still fresh in their memory. An example of a frequent problem was inconsistency in the date of the survey as reported on the different sheets (e.g. description of site conditions, determination of species). Sometimes this was due to operator errors (e.g. an incorrect date generated accidentally by dragging a spreadsheet cell) or poor definition of the reference date (e.g. confusion between date initially planned according to the protocol, the real date of the field survey, the date the sample was sent to experts for determination, the date of the determination). In addition, the time was often recorded without reference to the time zone or to summer/winter time. This information is often perceived as useless detail, but it becomes key when the data set is associated with information collected by automated sensors, for example, weather stations or remote sensing data. The difference between a survey not being conducted because of bad weather, for example, or being conducted but with no species observation was often not properly recorded, generating uncertainty that can affect analysis at a later stage. Another frequent problem was linked to plain text formats (e.g. csv or txt files, or dbf files associated with shapefiles). As the reference encoding is not stored as part of these files, moving from one system to another (with implicit assumption that the encoding matches local settings) corrupts the files, for example, by changing all special characters.

#### Data harmonisation

To process data from the four parks collected using the same protocol, we had to work on harmonisation to ensure interoperability between the four repositories. Although the data collection protocol was the same and the data were supposed to be already harmonised, many differences were found because the protocol was applied in slightly different ways according to different interpretations of the guidelines. This includes, for example, inconsistent recording of coded information and different procedures to report data and manage unforeseen situations. Based on the data control and import into the database, critical points were identified and data collection protocols optimised accordingly.

#### Internal technical skills

Finally, a key issue was the availability of internal technical skills. Hardly any of the technicians had any specific prior knowledge of databases and data management. However, some had a solid scientific background that allowed them to quickly acquire the necessary skills through short courses and by working on the data with initial support from an external consultant (training on the job). The use of a database was proved to be advantageous for the data analysis phase, for example, in extracting legally required information such as institutional reporting, environmental impact assessments, drafting of national conservation plans, and production of scientific outputs. This motivated park experts to learn the new tool.

## Conclusions

In this section, our findings are summarised in 10 lessons learned which we believe can help to stimulate discussion on how protected areas can improve collection and management of data that can be preserved in the long term and potentially reused. Some lessons are not new to ecological research and the recommendations provided may be taken for granted by experienced data managers. Nonetheless, they are not trivial for many researchers and conservation managers (Rüegg et al., 2014). The recommendations provided are at the core of unresolved issues that must be properly addressed by the administrations of the protected areas and by their funding institutions. We believe that shining a spotlight on common issues in protected areas, through a bottom-up approach, is an important contribution to the ongoing discussion on data management and sharing in ecology.

### Data quality control is critical

Using and sharing data without proper quality control can mislead the interpretation of ecological processes, hampering the success of management and conservation efforts. It is often assumed that data are collected and stored correctly, and controls are limited to automated checking of the logical correctness of the information. During our work, it emerged that this was not the case in most data sets processed. Careful screening of data is always necessary and should not be neglected because once data are archived, many errors or inaccurate information may be hard to detect. It is better to run quality controls immediately after collecting the data, involving both formal checks on the completeness and correctness of the information, and biological considerations to verify that the information is reliable. This requires data handling expertise, biology skills, and a good knowledge of the data collection protocols used. This process can be optimised by using appropriate data recording and processing tools and by collecting data with a certain degree of redundancy to facilitate controls. Data collection does not end with fieldwork; it extends to deskwork for data curation and storage. While this requires additional time and resources, it will not only lead to better data but also result in saving time and money in the long run. Adoption of data management plans (Michener, 2015b) is the best approach to achieve this. This is an established practice in the scientific field (Sutter et al., 2015) but not yet for many protected areas, where these plans must be tuned to the needs of entities operating in the long term and with a wide range of objectives (Pressey et al., 2015). Such plans can work as synthetic guides indicating responsibilities, coordinating all experts involved, and allowing for compatibility over time and between surveys.

The scientific community can help this process in many ways. While scientific literature offers a complete and convincing general framework for good scientific data management, it presents very few practical examples of quality-checking procedures and protocols. Although some practical courses and materials are available on the web, the existing best practices (e.g. Costello & Wieczorek, 2014; Michener, 2015b; Whitlock, 2011), technical guidelines (e.g. Borer et al., 2009; British Ecological Society, 2019), and training material (e.g. Data Carpentry, https://datacarpentry.org/) are focused on the specific requirements of scientific projects that may be different from those of other contexts including monitoring and management in protected areas.

### Recovery of historical data sets is urgent

The long-term surveys of wildlife, vegetation, and abiotic factors established by protected areas as part of continuous monitoring programmes, together with the huge number of occasional data collected over decades, are a potential goldmine of data. However, they are often not available in a ready-to-use form, preventing them from being fully harnessed. To be fully appraised, these data sets must be digitised, quality checked, documented, and structured into information systems. Ideally, at the end of this process, data should be made discoverable and accessible, according to FAIR principles, in internationally recognised repositories that also offer guidelines on data standardisation. This work on historical data sets is urgent because as time goes by, memory and records are progressively lost. It becomes difficult to retrieve complete information, transform it into digital format, check incongruences, and correct transcription errors, possibly increasing the risk of losing data completely. Extraordinary funds must be dedicated to this activity, which can also be conducted with the support of external experts, as staff hours are frequently fully allocated to ongoing management and monitoring activities.

### Appropriate tools must be used to manage the data

The quantity and complexity of data collected in protected areas, especially given the increasing use of monitoring sensors, call for specific tools and an adequate infrastructure to properly screen, store, and make data available in a multi-user, distributed environment. While for very small, individual research projects, it is still in some cases possible to use flat files such as spreadsheets, comma-separated values, and shapefiles, we believe that for many of the protected areas, the use of a centralised relational database in a secure and frequently backed-up server is the best option. Even simple data sets that could be handled individually with spreadsheets should go into a centralised repository. Here, rules can be set to enforce data integrity, users can access the information with a controlled permission policy, no multiple versions of the same data sets are permitted, and long-term preservation is guaranteed. In turn, this can increase research efficiency and positively impact conservation policies. Lastly, we recommend that the tools used are based on open-source software to ensure implementation of standards and interoperability with other systems. The use of an open software ecosystem guarantees maximum flexibility and ensures efficient use of resources. The setup, development, and maintenance of a database can be challenging tasks for protected areas because of the advanced technical skills required. The use of resources shared among protected areas and the use of external support can be effective solutions.

### Data management skills are needed

Effective data quality checks, integration of historical data, and use of advanced platforms all require specific management expertise that is often not or only partially available among staff of protected areas. It may be challenging for teams without experts with this expertise or a similar background to acquire these skills (Hunt et al., 2015), but it is a challenge that can be addressed with a combination of training, particularly training on the job (Kaplan et al., 2021; Petters et al., 2019), and time to systematically dedicate to this activity. Demonstrating the advantages of improved data management emerged as a key factor for motivating staff to be proactively involved in the training. During the project, we demonstrated how turnover of experts who are not permanent staff may be a big issue, as all the experience and expertise acquired are quickly lost. At the same time, in most cases permanent staff are overwhelmed with tasks that prevent them from devoting much time to an additional activity. Our experience suggests that protected areas can rely on external technical support for specific activities and outsource infrastructure development and maintenance. Nevertheless, in the medium to long term at least one internal staff member needs to have the skills and time to dedicate, even non-exclusively, to management of the data and to exploiting the opportunities offered by the use of a centralised repository. The staff involved in data collection may have only basic knowledge of how to handle data correctly and be responsible for curation of their own data sets. As for the infrastructure, sharing training and data management experts among protected areas is also an effective solution, as demonstrated by our project. Finally, academic curricula in ecology should include more information on data management, because individuals with skills in both domains are still rare. We are convinced that this will result in more active participation by protected areas in data sharing initiatives and in the adoption of FAIR principles.

### Data documentation is not optional

Producing good documentation and metadata ensures that data can be understood and used in the long term. It is not complex to document data but it requires time. This is of key importance for protected areas because of their long-term perspective, the inevitable rotation of technical staff, and evolution in data collection protocols, methodologies, and technologies. We experimented with problems related to missing or incomplete documentation while processing historical data sets, which led us to suggest that it is more efficient to document data as soon as they are generated. We encourage protected areas to compile and share metadata in a standard format to help others to discover their data. This is important for data reuse, but in some cases it is hard to offer a complete description of all the information needed for correct interpretation of the data. It is difficult to formalise the multiple connections between biotic and abiotic factors, collection protocols, local conditions, and the effect of land management that are typical of complex surveys (Zimmerman, 2008). This can lead to incorrect conclusions especially when used in automated processes as in the case of machine learning (for example, presence/absence of a species over the course of the year, which are not related to ecological variations but rather to population management). For this reason, in addition to compiling the best possible metadata, we strongly recommend that experts from the protected areas are also involved in analyses of their data when taking decisions for conservation goals and for scientific outputs. This can also act as a strong incentive to push for data sharing.

### Shared data collection protocols are a big opportunity

We found that the use of a common protocol for data collection within the four parks and the creation of interoperable databases with harmonised information opened new perspectives in the potential use of data. It saved huge amounts of time combining data from different sources at a later stage and allowed for consistency between them. These collaborative surveys offered the opportunity to compare different environmental gradients and their spatial distribution and should be encouraged as much as possible. Centralised institutions (such as regional, national, and international authorities) coordinating and supporting protected areas should be made aware of the importance of shared protocols and common projects for environmental monitoring across different protected areas, and the opportunities that they can offer. They should be encouraged to invest in these, possibly adhering to international standardisation initiatives. This, in turn, would allow data collected during ordinary surveys to achieve consistency similar to research-like data types, thereby supporting their usability for scientific purposes, as targeted by international initiatives such as European Long-Term Ecosystem Research (eLTER, https://elter-ri.eu/), Long-Term Ecological Research (LTER, https://lternet.edu/), and the Global Biodiversity Information Facility (GBIF, https://www.gbif.org/; Hobern et al., 2019).

This process would benefit from wider adoption of data and metadata community standards, but there are several barriers to this. Standards are not yet established in many ecological domains (e.g. Campbell et al., 2016) or are available for only a limited set of ecological information (Hackett et al., 2019). Darwin Core (Wieczorek et al., 2012) is the main reference in biodiversity, particularly for species presence and distribution, but it only partially addresses the complexity of most ecological studies carried out in protected areas (Costello & Wieczorek, 2014; Lynch, 2008). Efforts to extend it beyond species occurrence are still ongoing. Even more importantly, standards are often complex for non-scientific staff to adopt (Alves et al., 2018). Additional work is also needed on publishing and exchange of protocols (including those for data collection), best practices, and data management models that are tuned to the needs of protected areas and their staff.

### Data collected by partners should be stored in-house

A major problem often linked to the lack of an internal repository and clear protocols is the management of data collected within protected areas by projects that are carried out by partner institutions, for example, researchers working for universities or research centres within specific scientific projects. In the four parks, this often resulted in information collected by partners that did not remain available to the parks after the projects ended. Each data set collected, even by external research institutes, should have a data management plan that provides for its inclusion in a database managed by the protected area itself, and clearly defines criteria and formats.

### Data sharing is the final step in a long journey

The value of data increases when all researchers within a community can share and interact with each other’s knowledge (Chamanara & König-Ries, 2014; Michener & Jones, 2012). Data sharing is a crucial step in advancing science and providing answers to global environmental questions raised by society (Roberts & Moritz, 2011). Many papers have recently advocated data sharing and have discussed how this can be achieved (e.g., Enke et al., 2012; Parr & Cummings, 2005; Reichman et al., 2011). This is having a virtuous effect in the scientific community where many shared ecological data infrastructures are available, such as GBIF (Edwards, 2004), the German Federation for Biological Data (GFBio; Diepenbroek et al., 2014), the Ocean Biogeographic Information System (OBIS; Grassle, 2000), and Movebank (Kays et al. 2022). However, proper data curation to verify data quality is sometimes partially overlooked (Maldonado et al., 2015; Zizka et al., 2020) or limited to formal and automated controls. There are initiatives at international and global level to support the implementation of FAIR principles, but they often focus on the perspective of scientific institutes. We believe that this top-down approach undermines the proactive involvement of protected areas. Currently, there is too wide a technical gap for practitioners working with spreadsheets to deal with issues such as using controlled vocabularies (König et al., 2019) and presenting standardised data in machine-readable formats. For many of them, numerous preliminary stages are needed before data can be shared: acquisition of internal capacities, setting up of a solid infrastructure, recovery of historical data sets, in-depth quality check, and documentation. The value of long-term ecological data can be realised only if these are of the highest quality (Anderson et al., 2020; Michener, 2016). Based on our experience, we think that forcing sharing of data that are not ready can bring a high level of uncertainty about their reliability. Pushing for data sharing is legitimate but it must be clear that it is the last step in a long process and that technical support, resources, and time are needed to get there (Michener, 2015a). In addition, decisions on data access policy in national parks, and in most protected areas, are often not taken by those who manage them, and the traditional lever (citation mechanism) used to stimulate the openness of scientific data (Reichman et al., 2011) may not be equally effective. In this case, opening up data belongs to a different domain and pressure should be generated through raising awareness of public opinion to have an impact on political decisions. More can be done to incentivise the integration of databases from protected areas into the growing number of international research data infrastructures, but any integration model must consider the strong institutional nature of such repositories.

### Good data management means less work, not more

The administrations of protected areas must become aware of the need to take a leap forward in data management, given that the variety, complexity, and amount of data that national parks, and protected areas in general, need to manage is likely to grow exponentially in the future. This is emphasised by the commitment of many countries to enhance the accessibility of information to support decision-making (as in the Kunming-Montreal Global Biodiversity Framework) and the need to avoid waste of data in ecology (Purgar et al., 2022). Strong motivation is needed to overcome the reluctance to change work habits and to cover the initial costs of workflow and software updates, including hiring expert advisory staff and training personnel (Urbano et al., 2010). To raise awareness among the managers of protected areas of the importance of data management, we suggest the best argument is to show that better data management means that they can achieve better performance in their mandate of conservation, improve their impact on science, and simplify and speed up use of the data when approached by decision makers who need data as basis and justification for their political decisions (BID-REX, 2019). During the project, we tested concrete examples of greater efficiency and speed when extracting data in a ready-to-use format to produce required outputs. These include institutional and systematic reports related to habitat and species monitoring within the framework of the European network of protected areas Natura 2000 (EEC, 1992); ad hoc data extraction for legal obligations associated with environmental impact assessment studies inside protected areas; and data requests from the Ministry of the Environment or the Italian Institute for Environmental Protection and Research (ISPRA) for national reports (e.g. national report on the impact of wolves on livestock activities, national conservation plans for galliformes, national database on ungulates). Other examples not related to institutional activities are the inter-annual analysis of biodiversity patterns common to the parks, taking into account temporal and spatial gradients, which also involves production of scientific output (e.g. Chamberlain et al., 2020), and the automatic or semi-automatic integration of data into collaborative infrastructures—such as Ornitho, the Italian platform for ornithological data sharing (https://www.ornitho.it/), and Euromammals (https://www.euromammals.org), a pan-European network for data and knowledge sharing in movement ecology (Urbano & Cagnacci, 2021).

### There is no free lunch—specific funds are needed

Protected areas need to improve data management to improve decision-making, and this cannot be delayed much longer. Alongside a change in mentality, additional resources are needed for data management activities, to set up infrastructures and to train staff (Mons, 2020). This will bring major benefits, and agencies that finance protected areas at local level (e.g. municipalities, provincial governments) and national level (e.g. environmental ministries), while understandably interested in short-term success, must recognise the usefulness of long-term activities that involve structural rather than one-off, large investments. In this respect, international institutions, for example, the European Union, particularly the Natura 2000 network of protected areas (EEC, 1992) and the Europa Biodiversity Observation Network (EUROPABON, https://europabon.org/), can play a major role in pushing this process. Resources should cover the time that staff need to devote to data management activities, and where necessary internal or external specific technical skills for development and maintenance of the data infrastructure.

### Supplementary information

ESM 1 (DOCX 18 kb)

## Data Availability

Data sharing is not applicable to this article as no data sets were generated or analysed during the current study.
