# Correction to: Enhancing biodiversity conservation and monitoring in protected areas through efficient data management

**DOI:** 10.1007/s10661-024-12886-7

**Published:** 2024-07-15

**Authors:** Ferdinando Urbano, Ramona Viterbi, Luca Pedrotti, Enrico Vettorazzo, Cristina Movalli, Luca Corlatti

**Affiliations:** 1 European Commission, Joint Research Centre (JRC), Ispra, Italy; 2 Gran Paradiso National Park, Via Pio VII 9, 10135 Torino, Italy; 3 Stelvio National Park, Via De Simoni 42, 23032 Bormio, Italy; 4 Dolomiti Bellunesi National Park, Piazzale Zancanaro 1, 32032 Feltre, Italy; 5 Val Grande National Park, Piazza Pretorio 6, 28805 Vogogna, Italy; 6 Chair of Wildlife Ecology and Management, University of Freiburg, Tennenbacher Straße 4, 79106 Freiburg, Germany

**Correction to: Environ Monit Assess (2024) 196:12**

10.1007/s10661-023-11851-0

The original version of this article unfortunately published online with error in the copyright holder name.

The copyright holder for this article was incorrectly given as © The Author(s) 2023 but should have been © European Union and Ramona Viterbi, Luca Pedrotti, Enrico Vettorazzo, Cristina Movalli, Luca Corlatti 2023.

The original article has been corrected.

