# Supplementary material for: Enhancing biodiversity conservation and monitoring in protected areas through efficient data management
Source: Environ Monit Assess. 2023 Dec 5;196(1):12. doi: 10.1007/s10661-023-11851-0 (PMC10697885; doi:10.1007/s10661-023-11851-0)
Supplement: Supplementary file 1 — (DOCX 18 kb) [file 10661_2023_11851_MOESM1_ESM.docx]

# Additional Material

Description of the four national parks involved in the project

**Gran Paradiso National Park**

The oldest national park in Italy, the Gran Paradiso National Park, was instituted in 1922, for the conservation of Alpine ibex (*Capra ibex*). The main mission of the protected area is the conservation of the fauna and flora and the preservation of the geological formations in the area, as well as the beauty of the landscape for the present and the next generations. The park covers an area of more than 71 000 hectares surrounding the Gran Paradiso Massif (4 061 m asl), set at the centre of the protected area. It includes five valleys, three of which are in Aosta Valley and two in Piedmont, including 13 municipalities. The subalpine, alpine and nival planes are the most represented environments and cover most of the area: the average elevation of the territory exceeds 2 400 m asl ranging from 800 m to 4 061 m asl. This variability of altitudes, geology and climate corresponds to a very high level of animal and plant biodiversity. The park has its own surveillance service that monitors the protected area, constituted by park wardens, since 1947. The presence of this service allowed the beginning of many different monitoring projects, focusing on the presence and distribution of Alpine ungulates (Alpine ibex *Capra ibex* and chamois *Rupicapra rupicapra*), which now represents one of the longest time series available (1956-present) on mountain ungulates. In more recent years research and monitoring activities have been broadened considerably and now take an interest the eco-ethology of different species, e.g. Alpine marmot *Marmota marmota*, Alpine chamois and Alpine ibex as well as the ecology of freshwater ecosystems and the monitoring of biodiversity. Thanks to the collaboration between different universities and the scientific and surveillance services of the park, most of the research activities are established as long-term research projects with the aim of monitoring the response of flora and fauna to environmental changes.

**Stelvio National Park**

The Stelvio National Park was founded in 1935 with the aim of conserving natural resources, favouring socio-economic local development and promoting tourist fruition. It extends over some 135 000 hectares, across the region of Lombardy and the Autonomous Provinces of Trento and Bolzano, and encompasses the Ortles-Cevedale massif, in the south-western Alps. A vast altitudinal range, between 700 m and 3 900 m asl, as well as a complex combination of sedimentary and metamorphic deposits have generated a high diversity of habitats, representative of Alpine biodiversity. From 1935 to 1995, the SPN was administered by the State Forest Corps (Corpo Forestale dello Stato, CFS). In 1996, the park became a Consortium managed by several local administrations. This form of governance lasted until 2016, when the Stelvio National Park was split into three local administrations (Lombardy, Trento and Bolzano), characterised by marked cultural and socio-economic differences. The management of monitoring and conservation activities of natural resources, however, has remained unitary. The recently computerised historical archive includes annual data on the distribution and abundance estimates of key animal species (birds, mammals) collected by park rangers, as well as information concerning patrol activities, animal reintroductions and forestry. With the foundation of the Consortium, the scientific department has initiated annual standardised monitoring of ungulates, galliformes and large raptors, and has promoted several medium- and long-term research and monitoring activities on habitats, floristic and animal species.

**Val Grande National Park**

The Val Grande National Park, first established in 1992, was expanded in 1998, and extends over 14 598 ha within the Province of Verbano-Cusio-Ossola, in Piedmont, Northern Italy. The foundation of the protected area was aimed at preserving one of the largest wilderness areas of the Alps, as most of the park’s territory is devoid of human settlements. The park includes the Val Grande Nature Reserve (an integral and biogenetic nature reserve of 973 hectares) and the Monte Mottac Nature Reserve (an oriented and biogenetic nature reserve of 2 410 hectares), both established in 1971. The vicinity of the Lake Maggiore, and the elevation gradient spanning between 500 m and 2 300 m asl, favour the presence of a high floristic richness. Forest vegetation is dominated by mixed broad-leaved woods, while conifer woods are limited in extension, and Alpine meadows and rocky vegetation environments dominate above the treeline. The park hosts various wildlife species, typical of montane and Alpine habitats. Since its foundation, the park has conducted research and monitoring activities on several aspects, including standardised counts of ungulates species, surveys of aquatic wildlife, analyses of biodiversity evolution from an historical and socio-economic perspective, and, more recently, standardised surveys of large raptors and animal biodiversity along altitudinal gradients.

**Dolomiti Bellunesi National Park**

The Dolomiti Bellunesi National Park was founded in 1993 to protect an area very rich in biodiversity. It is the easternmost of the 4 Italian Alpine national parks, extending over 31 000 hectares in the southernmost part of the Dolomites. In 2009, it was declared a Natural World Heritage site by UNESCO. The park is home to around a quarter of the entire Italian flora and hosts many animal and plant species that are either endemic or at the limits of their distribution range. The biological richness that characterises the area is determined by various and concomitant factors: a vast altitudinal range, between 400 m and 2 565 m asl which, alongside a great lithological diversity and an articulated orography, determines the presence of diversified habitats and peculiar microclimatic situations; the geographical position, which made it a massive refuge during the Quaternary glaciations and a ‘biogeographical crossroads’ between the Alpine and Illyrian areas. Since its foundation, the park has initiated research and monitoring activities to collect information about species occurrence, distribution and conservation status. Since 1995, standardised counts of grouse and ungulates have been carried out, and distribution atlases of avifauna and herpetofauna have been published. The park has up-to-date checklists of flora and fauna, and in recent years has begun the standardised monitoring of animal and plant species included in the Habitats Directive, to assess their status and the effectiveness of the conservation and active management of the habitats.
